# Functional human *GRIN2B* promoter polymorphism and variation of mental processing speed in older adults

**DOI:** 10.18632/aging.101228

**Published:** 2017-04-24

**Authors:** Yang Jiang, Ming Kuan Lin, Gregory A. Jicha, Xiuhua Ding, Sabrina L. McIlwrath, David W. Fardo, Lucas S. Broster, Frederick A. Schmitt, Richard Kryscio, Robert H. Lipsky

**Affiliations:** ^1^ Department of Behavioral Science, University of Kentucky, Lexington, KY 40536, USA; ^2^ Department of Molecular Neuroscience, the Krasnow Institute for Advanced Study, George Mason University, Fairfax, VA 22030, USA; ^3^ Sanders-Brown Center on Aging, University of Kentucky, Lexington, KY 40536, USA; ^4^ Department of Neurology, University of Kentucky, Lexington, KY 40536, USA; ^5^ Departments of Statistics and Biostatistics, University of Kentucky, Lexington, KY 40536, USA; ^6^ Department of Neurosciences, Inova Neuroscience Institute, Inova Health System, Falls Church, VA 22042, USA

**Keywords:** short-term memory, cognitive aging, working memory retrieval, NMDA receptor, *GRIN2B* gene, functional polymorphism

## Abstract

We investigated the role of a single nucleotide polymorphism rs3764030 (G>A) within the human *GRIN2B* promoter in mental processing speed in healthy, cognitively intact, older adults. *In vitro* DNA-binding and reporter gene assays of different allele combinations in transfected cells showed that the A allele was a gain-of-function variant associated with increasing *GRIN2B* mRNA levels. We tested the hypothesis that individuals with A allele will have better memory performance (i.e. faster reaction times) in older age. Twenty-eight older adults (ages 65-86) from a well-characterized longitudinal cohort were recruited and performed a modified delayed match-to-sample task. The rs3764030 polymorphism was genotyped and participants were grouped based on the presence of the A allele into GG and AA/AG. Carriers of the A allele maintained their speed of memory retrieval over age compared to GG carriers (*p* = 0.026 slope of the regression line between AA and AG versus GG groups). To validate the results, 12 older adults from the same cohort participated in a different version of the short-term memory task. Reaction times were significantly slower with age in older adults with G allele (*p* < 0.001). These findings support a role for rs3764030 in maintaining faster mental processing speed over aging.

## INTRODUCTION

Cognitive aging and onset of dementia initially affect short-term memory, whereas long-term memory is largely retained. A consistent finding in the cognitive aging literature is the general slowing in mental processing speed, manifesting as increased reaction times during cognitive tasks [1]. Compared to young adults, middle age and older adults exhibit slowing in mental processing speed during working memory tasks [2]. Additionally, neural responses in the visual cortex in response to visual stimuli are delayed in older individuals [3]. Multiple brain imaging studies have demonstrated that to accomplish a simple cognitive task, older adults require the activation of additional brain networks than young adults, possibly as a compensatory mechanism [4, 5, 6, 7].

Despite progress in cognitive and brain aging research, the molecular and cellular basis for inter-individual differences in brain aging and why some older adults retain their sharp mind, is not yet well understood. Significant relations of genetic factors and individual differences in working memory performance of healthy, cognitively intact, older adults have been demonstrated [8]. These previous studies implicated the contribution of genetic variation in dopaminergic/noradrenergic genes in cortical activity, and to inter-individual variation in working memory and decision behavior. Other neuro-transmitter systems are also important in memory performance, but have been understudied. Of particular interest is the N-methyl-D-aspartate (NMDA) receptor, an ionotropic glutamate receptor central in synaptic plasticity underlying learning and memory formation [9,10]. Dysregulation of the NMDA receptor is used to model dementias and Alzheimer's disease (AD) [11,12] in rodents. A mouse model of AD demonstrated that soluble amyloid beta oligomers can interact with the pore forming GluN2B subunit to induce hyperexcitability of the NMDA receptor, blocking long term synaptic potential and inducing excitotoxicity [13].

A genome wide association study (GWAS) identified two single nucleotide polymorphisms (SNPs) in introns of the GRIN2B gene (*GRIN2B*), encoding the GluN2B subunit, that were significantly over-represented in patients with AD [14]. This was the first finding of a genetic association between *GRIN2B* and an endophenotype. The GluN2B subunit is expressed in neurons in the hippocampus and forebrain. Genetic and pharmacological animal studies demonstrated that genetic ablation of *GRIN2B* or pharmacological inhibition of GluN2B resulted in decreased memory formation and consolidation [15, 16]. In contrast, transgenic animals overexpressing GluN2B displayed superior learning and memory performance compared to wild types [17]. In addition, decreased levels of GluN2B protein are correlated with advanced age in the hippocampus of aging mice [18] and decreased levels of GRIN2B mRNA during Alzheimer's disease progression [19]. Interestingly, memory in aged mice can be rescued by enhanced expression of GluN2B [20], suggesting an approach for developing cognitive enhancers.

Transcription of *GRIN2B* is controlled by several proteins including members of the E26 transforming-specific (ETS) transcription factor family [21, 22, 23], cyclic AMP response element binding protein (CREB), and nuclear factor kappa B (NF-κB). The ETS transcription factor family is characterized by a central 5′-GGA(A/T)-3′ DNA binding site with flanking sequences that are thought to contribute to the ETS binding specificity [24]. At present, 28 members of the ETS family, subdivided into 12 subgroups, have been identified in humans [24, 25]. Researchers have used combined chromatin immunoprecipitation, microarrays, or massively parallel sequence analysis to identify a subset of ETS transcription factors that preferentially bind the 5′-CCGGAACT-3′ eight nucleotide sequence in various cell types [24,26]. In particular Elk-1, known for its role in pro-apoptosis and pro-differentiation in neurons, has been shown to be involved in learning and memory [27, 28].

Here, we investigated the role of the single nucleotide polymorphism (SNP) rs3764030 within the *GRIN2B* promoter region in working memory retrieval in healthy, cognitively intact older adults. We demonstrate in two experiments that the rs3764030 G>A SNP forms a *de novo* ETS binding site resulting in a gain-of-function phenotype, which is associated with maintained mental processing speed over aging.

## RESULTS

### The SNP rs3764030 A allele creates a novel *in vitro* ETS transcription factor binding site

**Figure 1:**
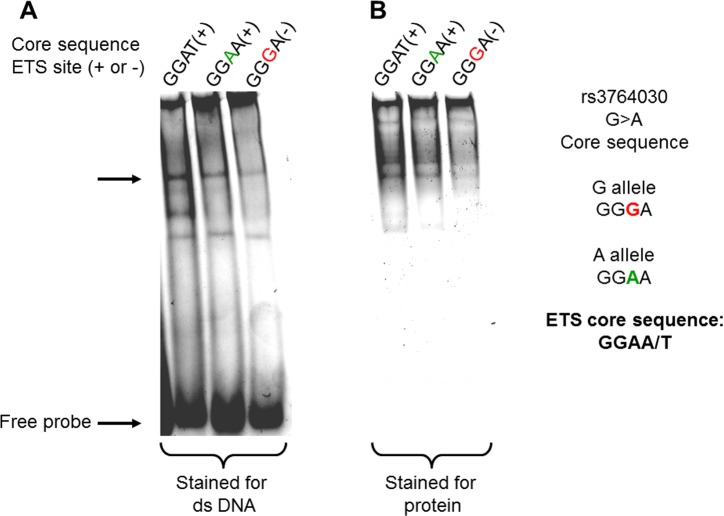
Binding of recombinant Elk-1 protein to dsDNAs containing the rs3764030 variant A allele from the human GRIN2B gene promoter Double-stranded DNA targets (Methods) were incubated with1 μg recombinant human Elk-1 protein on in EMSA buffer. DNA-protein complexes were electrophoresed through 6% polyacrylamide gels in HEPES buffer pH 6.3, without polydIdC. The gel was then stained with the SYBR Green to visualize DNA (**A**) and SYPRO Ruby for protein bound to DNA (**B**). The stained gel was scanned in G:BOX (Syngene, US) for imaging. Elk-1 bound to DNA is indicated by the arrow.

We tested the hypothesis that a SNP (rs 3764030, G>A) within the promoter region of *GRIN2B* gene produces a *de novo* ETS family transcription factor binding site. An *in vitro* DNA binding assay was developed using purified Elk-1, an ETS family member expressed in the central nervous system [28], and DNA targets containing either (1) the consensus ETS core binding site (GGAA/T), (2) the ETS binding site identified in the *GRIN2B* promoter region (GGAA, A allele), or (3) without the ETS binding site (GGGA, G allele). The results in Figure 1 show that the A allele DNA probe bound recombinant Elk-1 to a greater extent than the G allele DNA probe (Panel A). When the same gel was stained for protein, it was readily apparent that the A allele DNA probe had more protein associated with it than the G allele DNA probe (Panel B).

### NMDA receptor activation in N2a cells transfected with rs3764030 A allele plasmid induced greater reporter gene activity than transfection with rs3764030 G allele

**Figure 2:**
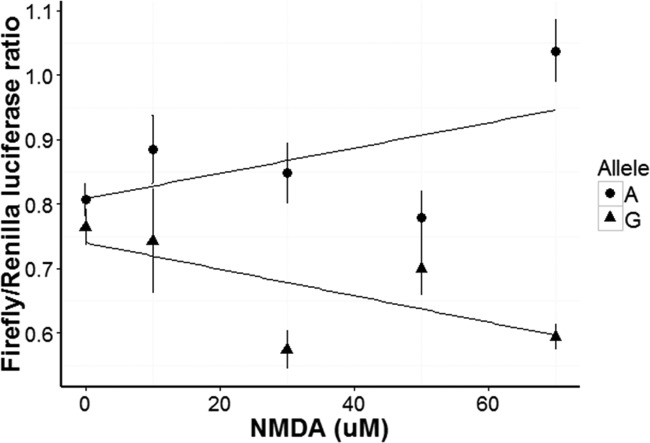
Concentration dependent response of human *GRIN2B* promoter in N2a cells carrying either the A or G alleles of rs3764030 to NMDA Luciferase reporter gene plasmids were constructed in pGL 4.10 (to produce Firefly luciferase, Promega, US). Luciferase reporter plasmids and the pGL4.75 plasmid (to produce Renilla luciferase as a reference) were co-transfected into retinoic acid differentiated N2a cells and assayed for Methods). After 24 hours of transfection, the cells were incubated for six hours with 0, 30, 50, 70 or 90 μM NMDA for transcription factors binding and then replaced with complete media (with 10% FBS) and cultured for an additional 40-44 hrs. Data are presented as means ± SE.

Murine neuron-like N2a cells, which express Elk-1 [29] and NMDA receptors [30], were used to study the function of the SNP rs3764030. Differentiated cells were transfected with plasmids containing either the rs3764030 A or G allele and firefly luciferase and a second plasmid containing Renilla luciferase. Transfected cells were stimulated with different concentrations of NMDA (0, 10, 30, 50, 70 μM) and luciferase activities measured. A dose-dependent increase in Firefly/Renilla luciferase activity was seen for the A allele (Fig. 2). N2a cells transfected with the SNP rs3764030 G allele did not respond to NMDA. These data suggest that the A allele produces an NMDA receptor gain-of-function phenotype.

### Participant characteristics

A description of the 28 healthy aged adults in Experiment 1 stratified by rs3764030 genotype is provided in Table 1.

**Table 1:** Participant characteristics for Experiment 1

	A carrier	GG	P-value
Age	75.4 ± 5.5	73.5 ± 6.0	0.39
Sex (M/F)	6/7	6/9	0.96
Education (Years)	16.1 ± 2.8	16.2 ± 2.6	0.91
MMSE	29.1 ± 0.86	29.2 ± 0.68	0.34
APOE ε4 Status (0/1)	11/3	11/2	0.93

Two-tailed P-values were obtained from ANOVA for continuous traits, or by chi-square tests, for categorical variables. APOE ε4 status: 0 = non-carrier, 1 = ε4 carrier.

Participants (n = 28, 15 GG vs 13 A allele carriers): allele frequencies met Hardy-Weinberg expectation based on frequencies observed in European populations.

When stratified by A allele carriers and GG genotypes, the groups did not differ based on age, sex, education, or *APOE* ε4 carrier status (Table 1). Both genotype groups had high cognitive function and had comparable mean scores of 29 on the Mini-Mental State Exam. (MMSE; Table 1).

### Experiment 1. Association of rs3764030 genotype with memory performance

**Figure 3:**
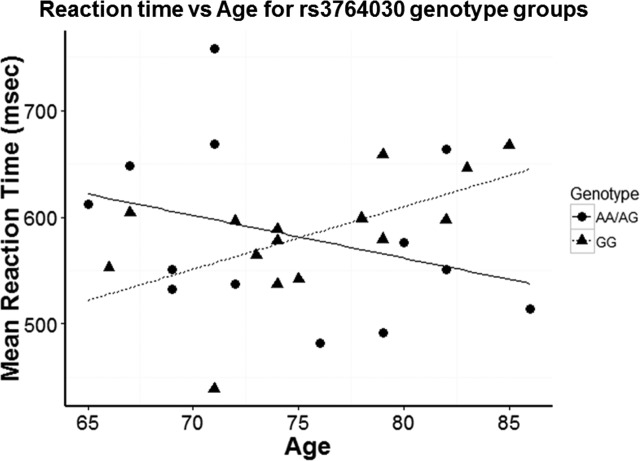
Reaction time in Experiment 1 participants with increasing age based on stratification by GG genotype and A allele carriers (AA and AG genotypes) group differences A significant difference in the slopes of the regression lines was observed for A allele carriers (−3.99 ± SE 3.47) versus the GG genotype group (5.84 ± SE 2.28) (*p* = 0.026). R^2^ for GG regression was 0.336 while R^2^ for A allele carriers was 0.107.

The NMDA receptor has been shown to be critical for learning and memory. We therefore investigated if the SNP rs3764030 in the *GRIN2B* promoter region would correlate with a memory performance phenotype. We hypothesized that aged individuals with the SNP rs3764030 A allele would perform better in a visual memory test than those with a homogenous G allele. Using the Delayed Match-to-Sample (DMS) task, we determined that there was a significant correlation between participants' age and reaction time when grouped based on SNP rs3764030 genotype. A positive correlation between age and reaction time was found in subjects with the GG genotype (Fig. 3). In contrast, subjects in the AA and AG genotype group (A allele carriers) had a negative correlation between age and reaction time (Fig. 3). The slopes of the regression lines differed significantly between subjects with and without an A allele (−3.99 ± SE 3.47 for A allele carriers vs 5.84 ± SE 2.28 for GG; t(24) = 2.37, *p* = 0.026). The negative slope of the regression line for A allele carriers was associated with improved performance, but was not significantly different from a flat slope (p = 0.275). This may be due to the dispersion from combining AA and AG genotypes. The R^2^ for GG regression was 0.336 while R^2^ for A allele carriers was 0.107, indicating greater variability in reaction time. In a different measure, mean reaction time was not significantly different between A allele carriers and the GG genotype group (A allele carriers: 583 ± 81 msec; GG 583 ± 54 msec; *p* = 0.490, Students' t-test).

### Experiment 2. Replication sample

To validate the results from the Experiment 1, we performed a second experiment using a shorter and slightly simpler version of the working memory task (See Methods section).

**Figure 4:**
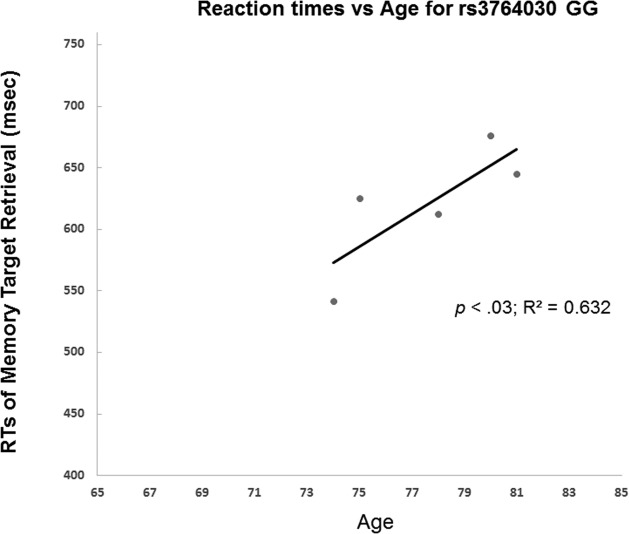
Reaction time in Experiment 2 participants with increasing age was examined in GG homozygotes

**Figure 5:**
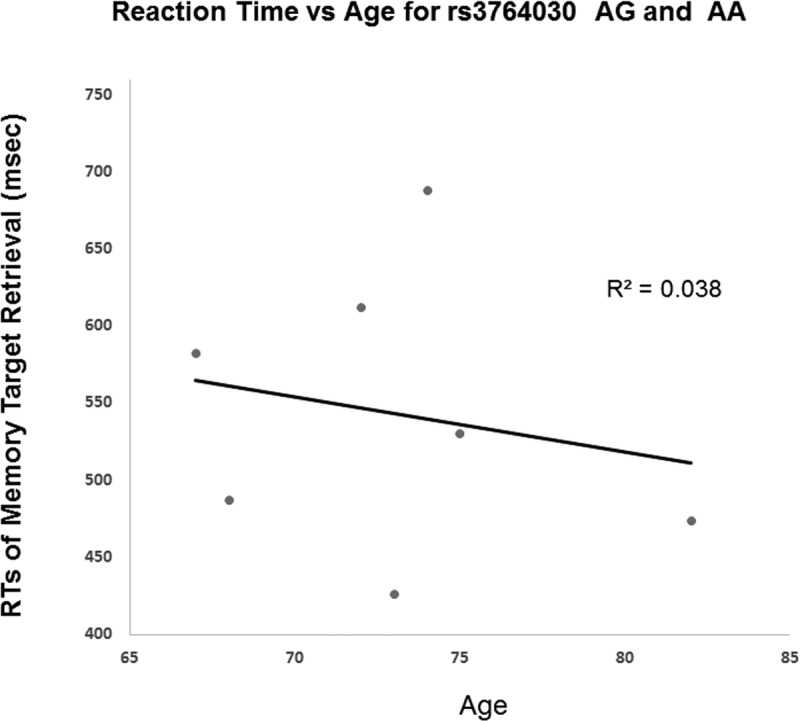
Reaction time in Experiment 2 participants with increasing age based on A allele carrier status (AA and AG genotypes) A significant difference in the slopes of the regression lines was observed for A allele carriers (−3.58 ± SE 0.21) versus the GG genotype (13.1 ± SE 0.20) (*p* < 0.001).

The results from Experiment 2 showed GG and A allele carrier did not differ in memory accuracy (on average 97%) of retrieval of the memory targets. Yet, they differed in reaction times in repeated retrieval of visual target held in working memory. GG indiviuals had a positive correlation between age and reaction times of 3^rd^ retrieval of memory target (Fig. 4). That is, individuals with the GG genotype had increased reaction times of memory retrieval with age. In contrast, A allele carriers (AA and AG genotypes) did not show such trend. The A allele carriers reactions times even decreased slightly with age (Fig. 5). The slopes of the regression lines were significantly different (−3.58 ± SE 0.21 for A allele carriers versus GG genotype 13.1 ± SE 0.20, t(8) = 58.5, *p* < 0.001). These results were consistent with those from Experiment 1.

## DISCUSSION

### Summary of results

We tested the hypothesis that individual differences in processing speed are partially determined by a SNP within the promoter of the *GRIN2B* gene. In two experiments, memory performance among A allele carriers, predicted better performance during aging. These findings in two groups of cognitively normal aging adults suggest that the A allele may confer a protective effect on one aspect of memory performance. Behavioral results showed that presence of the A allele was not associated with differences in mean reaction times. However, A allele carriers showed decreased reaction time with age compared with the GG genotype (p = 0.026 based on difference in slope of the regression line between GG and A allele carriers).

Changes in expression of the *GRIN2B* gene and activity of GluN2B subunit containing NMDA receptors are known to affect memory and cognition [17]. From the genetic perspective, variation in the human* GRIN2B* gene is known to be associated with normal and impaired memory [14,31], although the molecular and cellular mechanisms underlying these changes in memory performance are not well understood. In this study we determined a biological effect of the *GRIN2B* promoter SNP, rs3764030 G>A, showing that the A allele in transfected N2a cells, responded to NMDA agonism in a dose-dependent manner relative to the common G allele. In addition, we showed that the A allele was capable of binding the ETS transcription factor, Elk-1, *in vitro*. These observations were the mechanistic basis for supporting a gain-of-function gene variant and for combining genotype groups (A allele carriers vs non-carriers) for genetic association studies.

Evidence from neuroimaging studies indicates that working memory involves multiple cortical networks, e.g. the prefrontal, temporal cortex, and parietal, parahippocampus, and early visual occipital cortices

[32,33,34]. From animal studies and in humans, it is known that as the brain ages, the level of *GRIN2B* mRNA and GluN2B protein is reduced in certain brain regions [18,35,36,37,38]. While other SNPs, by individual or by haplotype-based analyses have supported the idea that *GRIN2B* has a role in gene expression and memory performance [39] and in AD [40], we think the current study is the first report of a SNP that affects *GRIN2B* mRNA levels by an activity-dependent mechanism. Knowing a functional polymorphism that increases levels of *GRIN2B* mRNA and possibly the number of GluN2B subunits suggests a way for protecting the brain from age-related cognitive decline. However, it should be noted that our study has limitations that will need to be addressed in future studies. First, the sample size was small, so these findings must be considered preliminary. We describe the association of one genetic variant on memory performance. SNP rs3764030 may interact with other variants of the* GRIN2B* gene or may be in linkage disequilibrium with other functional variants. An examination of linkage disequilibrium relationships among SNPs approximately 10 kb 5′ and 10 kb 3′ from SNP rs3764030 (European-American population of Northern and Western European ancestry, CEU, 1000 Genomes database) indicated that while linkage disequilibrium (LD) approached 1.0 based on D', the average R^2^ was low between rs3764030 and flanking SNPs (highest R^2^ value was 0.237)(Figure S1, Panel B, Supplementary Material, flanking SNP with available R^2^ within 20kb of rs3764030). Therefore, at least for individuals of Northern and Western European descent, the selected SNPs in this 20 kb region have a similar frequency as rs3764030 but have no better predictability than the functional variant we selected for analysis in the current study. Addressing the role of other genetic variants that regulate expression of the *GRIN2B* gene will require additional DNA sequence screening, mRNA expression analysis, and association analysis using larger cohorts.

Other questions relating to the role of ETS domain proteins in the brain are worthy of future exploration. The ETS domain transcription factor family has 27 known members that are expressed by different human cells. Our initial focus on Elk-1 was because it is expressed in the brain, among other tissues. Although these ETS transcription factors display binding site selectivity based on differences in nucleotide sequences that flank the core GGAA/T motif *in vitro*, convergent findings from different groups have shown that ETS family member proteins display overlapping DNA binding patterns between some family members *in vivo* [41,42]. In future studies, it will be of great interest to identify the ETS domain transcription factor that binds the A allele *in vivo*. This information may have implications in defining cellular changes that occur in the aging human brain that may then be used to predict vulnerability to neurodegenerative disorders such as AD.

## CONCLUSIONS

Our results from two experiments support the idea that presence of the A allele of SNP rs3764030 positively influenced an individual's mental processing speed during working memory indexing. Alternatively, individuals with the G allele, while having slower responses with increasing age, may have a stronger capability to suppress distractors. Either interpretation is consistent with the idea that changes in subunit stoichiometry of NMDA receptors confer distinct functional properties on memory. The grouping of A allele-genotypes for association analyses was justified based on reporter gene assays and gain-of-function ETS transcription factor binding to DNA. Future longitudinal follow-up in larger populations will bring insights of whether *GRIN2B* gene variation might be a potential indicator for cognitive reserve or may serve as a risk-factor for late onset dementia.

## METHODS

### Participants

Participants were drawn from a large aging cohort of over 400 older adults followed by the University of Kentucky (UK)-Alzheimer's Disease Center (ADC). All participants were enrolled from a prior existing longitudinal study on aging and brain health that collects demographic, health and neuropsychological data, and blood samples annually [43]. All research activities were approved by the University of Kentucky Institutional Review Board and all participants provided informed written consent prior to any research activities.

Individuals were not invited to participate in the study if they had a history of substance abuse, traumatic brain injury, major psychiatric illness, or illness affecting the central nervous system such as for example encephalitis or meningitis. Battery of Neuropsychological tests including the Mini-Mental State Examination were available for all participants in the present study. Subjects agreed to provide blood for genetic testing and to participate in annual clinical assessment and neuropsychological testing. In the first group (described later as Experiment 1), 28 (17 females) healthy, cognitively normal, right-handed, English-speaking, adults aged 65-86 participated in the current study.

To cross-validate the results of Experiment 1, 16 healthy older adults from the general cohort described above participated in the second experiment. Twelve of the 16 older adults had rs3764030 genotypes available for analysis together with memory performance data.

### Genotyping

Genomic DNA was isolated from whole blood as described previously [44]. For Experiment 2, genotypes for SNP rs3764030 were obtained from the UK-ADC samples. The older adults' blood samples were contributed to the Alzheimer's Disease Genetics Consortium, and genotype calls were returned to the UK-ADC. The genotyping data were extracted from the raw genotype file first and then merged with the behavioral performance data. The minor allele (A) was determined by calculating minor allele frequency based on the raw UK genotype files. SNP rs3764030 is detected in human populations of European and Asian origin. Details for genotyping reactions are given below.

### Primers

DNA, extracted from blood samples, were genotyped for the rs3764030 G>A SNP in the human *GRIN2B* promoter region using a 5′ exonuclease assay with allele-specific fluorescence detection probes. The rs3764030 SNP is located at nucleotide position 13,980,398 (Reference Sequence NC_000012.2) on Chromosome 12. The allele-specific detection probes were fluorescently labeled with either FAM or VIC at the 5′-end and linked to a non-fluorescent quencher (MGB) at the 3′-end.

Amplification primers

Forward: 5′-caaagcgtccccttcctaag-3′

Reverse: 5′- ctctcgtgtgcactctgtgg-3′.

The sequence of the allele-specific detection probes were:

G allele (shown as opposite strand, C): 5′-ttgattcgcgtgtccccc-3′

A allele (shown as opposite strand, T): 5′- ttgattcgcgtgttcccc-3′.

*APOE* variants rs429358 (C>T, Arg112Cys) and rs7412 (C>T, Arg158Cys) were genotyped as described previously [45].

### General method for performing 5′-exonuclease assay

Genotyping reactions (5 μl) were performed in 96-well plates containing 10 ng of genomic DNA, 0.5 μM of primers, 0.2 μM of probes, and 2.5 μl of Master Mix (Thermo). The reaction thermal cycle program consisted of 50°C for 2 min, 95°C for 10 min, followed by 40 cycles of 95°C for 15 s, 59 or 60°C for 1 min. End point amplification genotypes were determined using BioRad Sequence Detector. Genotyping accuracy was verified by re-genotyping at least 10% of randomly selected DNA samples using the same assay or direct sequence analysis. Genotyping accuracy was >99% and genotyping completion was 99%.

Genotypes were independently confirmed using direct sequence analysis with no discrepancies. Genotype frequencies met Hardy-Weinberg expectations in the study population.

### Molecular and cellular characterization of a functional *GRIN2B* SNP

#### Luciferase vector construction

To characterize potential effects of this SNP on transcription, we used PCR to amplify a 1.7 kb genomic region spanning from 1530 bps upstream of the human* GRIN2B* gene transcription start site into *GRIN2B* gene untranslated exon 4. Exon organization was based on RefSeqGene NG_031854.1 (Fig. S1, Panel A Supplementary Material). For PCR, the Forward primer: 5′ GAGCTCAAACCACTTCCTCCGGCTTC 3′ and the Reverse primer: 5′ CTCGAGGCCAACCTCTAGACGGACA 3′. Individuals with genotype GG and AA are selected to PCR amplify the 1.7kb region for cloning. The PCR product was inserted into the pGL 4.10 plasmid (Promega, Madison, WI, USA) that is a promoter-less vector containing a luciferase reporter gene. Four plasmid constructs were designed, one for each combination of alleles (A or G allele, positive and negative orientation), and the DNA strand transcribed: (1) positive orientation with allele A on the upper transcribed strand (A+ plasmid), (2) positive orientation with allele G (G+ plasmid), (3) negative orientation with allele A on the lower untranscribed strand (A- plasmid), and (4) negative orientation with allele G (G- plasmid). Orientations and alleles in the constructs were confirmed by direct sequencing with an ABI 310 genetic sequencer (Applied Biosystems, Foster City, CA, USA).

#### Functional NMDA receptor analysis

Murine N2a neuroblastoma cells (ATCC, Manassas, VA, USA) were grown in RPMI 1640 cell culture medium (Thermo Fisher, Pittsburgh, PA, USA) supplemented with 5% fetal bovine serum (FBS) in 5% CO_2_ at 37°C until cells were approximately 80% confluent. Cells were transferred into laminin- and poly-L-lysine coated 96 well cell culture plates (Corning 3904, Thermo Fisher Scientific, Pittsburgh, PA, USA) and grown in serum-free RPMI 1640 medium 24-48 hrs prior to transfection. Cell differentiation was induced by adding 1 μM retinoic acid (Sigma-Aldrich, St. Louis, MO, USA) to the culture medium for 24 hrs. Differentiated cells were co-transfected with one of the four pGL4.10 plasmid constructs encoding the Firefly luciferase reporter gene and the control pGL4.75 plasmid encoding the Renilla luciferase reporter gene using the Lipofectamine transfection reagent (Thermo Fisher) according to manufacturer's instructions. Transfected cells were activated by adding 0, 30, 50, 70, or 90 μ M NDA (Sigma-Aldrich) to the culture medium for 4-6 hrs to induce transcription factors. Solutions were replaced with RPMI 1640 medium containing 10% Fetal Bovine Serum (Atlanta Biologicals, Flowery Branch, GA, USA) for 40-44 hrs at 37°C in 5% CO_2_. Luciferase activities were measured using a Synergy 4 plate reader (Biotek, Winooski, VT, USA). Each NMDA concentration was tested three times for statistical analysis.

### Electrophoretic mobility shift assay

The electrophoretic mobility shift assay (EMSA) was used to monitor interactions between the ETS domain transcription factor Elk-1 and its DNA recognition sequence *in vitro*. The following sequences were used for producing a positive control double stranded DNA (dsDNA) targets based on Wei *et al*.,( 2010):

Positive control

Forward: 5′ ACGCTAACCGGATATAACGCTA 3′

Reverse: 5′ TAGCGTTATATCCGGTTAGCGT 3′

*GRIN2B* sequences were:

GRIN2B A allele

Forward: 5′ CATCTCCGGGGAACACGCGAA 3′

Reverse: 5′ TTCGCGTGTTCCCCGGAGATG 3′

GRIN2B G allele

Forward: 5′ CATCTCCGGGGGACACGCGAA 3′

Reverse: 5′ TTCGCGTGTCCCCCGGAGATG 3′

Complementary oligonucleotides were annealed with the Bio-Rad C1000 thermocycler (Bio-Rad, Berkeley, CA, USA) in annealing buffer (10 mM Tris-HCl (pH 8.0), 1 mM EDTA, and 50 mM NaCl ) with 1°C / 1 min decreasing from 95°C to 20°C. The annealed dsDNA was incubated on ice for 1 h with 1 μg recombinant human Elk-1 protein (Sigma-Aldrich, St. Louis, MO, USA) in EMSA buffer (50 mM HEPES pH7.5, 10 mM MgCl_2_, 5% glycerol, 1 mM DTT, 0.3% BSA, 1 mM EDTA). The EMSA was run in a 6% retardation gel (Thermo Fisher Scientific) using 95V for 80 min. The gel was stained with EMSA SYBR Green and SYPRO Ruby kit (Catalog E-33075; Thermo Fisher Scientific) according to manufacturer's instructions. The gel was scanned using an imaging G:BOX (Syngene, Frederick, MD, USA).

### Experimental paradigm to study visual working memory

#### Visual stimuli

Stimuli consisted of 216 two dimensional pictures of common objects taken from [46]. Each object was presented in white-black within a rectangular area of approximately, 8.3 by 5.8 cm. At the beginning of each memory trial, sample pictures were also presented with a 6.5 mm green border. Each picture group was normed for familiarity and complexity. All 216 stimuli pictures were transformed using Fourier analysis. They were named scramble pictures. For Experiment 1, scramble pictures were seen between trials, that was not the case for Experiment 2.

#### The modified delayed match-to-sample task

Experiment 1. The short-term memory task consisted of study and test phase. During the study phase, participants memorized 80 line drawing pictures until they reach 95% accuracy in immediate recognition.

**Figure 6:**
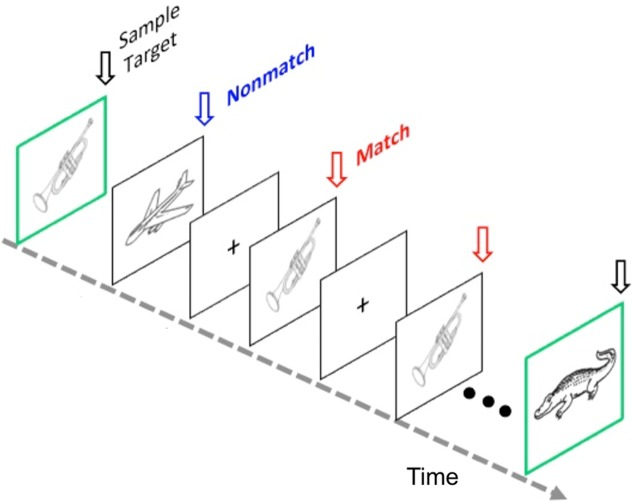
The modified Delayed Match to Sample (DMS) task Schematic shows a typical memory trial at the test phase of the task. Repeated retrieval of a target memory item (Match) occurs. Participants were first presented with a sample target object and then viewed test objects (target or distracters) in rapid succession for each memory trial. Subjects were instructed to forget the previous sample target object only when a new sample target object appeared. For Experiment 1, a study phase was included. During the study phase, participants memorized 80 line drawing pictures until they reach 95% accuracy in immediate recognition. Thus, half of the images were studied during the test phase. The sample target object was followed by 10 successive old and new distracter objects at a rate of 2 sec per picture. The DMS task consisted of 80 trials separated into eight blocks of 10 trials each. It took about 50 minutes to complete the task for each participant. The protocol for Experiment 2 used a shorter and simpler version of the task [47], which did not include a study phase, and total time is about 20 minutes for each participant.

Thus, half of the images were studied during the test phase. The test phase included 80 trials separated into 8 blocks of 10 trials each. Each trial began with the presentation of a sample target object for 2000 msec and was distinguished by a green border (Fig. 6). The sample target object was followed (ISI = 700 ± 100 msec) by 10 successive test objects with a stimulus duration of 2000 msec (ISI = 500 ± 200 msec). Each trial lasted 27.5 seconds. The test portion of each trial contained a pseudo-random presentation of target match and nonmatch objects where the match, a studied nonmatch, and a new nonmatch were presented three times each, resulting in nine of the ten test items in a trial. One additional ‘filler’ object was included in each trial to reduce the potential for subject expectancy and served either as a 4^th^ target (16.7 % of trials), 4^th^ studied nonmatch (16.7 % of trials), 4^th^ new nonmatch (16.7 % of trials), or a new nonmatch never previously shown (50 % of trials). None of the objects, whether serving as a target or nonmatch, were used in any subsequent trials. Across trials, stimuli from the three experimental conditions were equally distributed across all 10 serial positions. Figure 6 illustrates a memory trial.

Participants were told to hold the sample target object in mind and indicate whether the following 10 test objects were the same or different from the sample target. Assignment of hands to indicate a target match versus a nonmatch object was counterbalanced across right- handed subjects. Subjects were also instructed to forget the previous sample target object only when a new sample target object appeared in the next trial (Fig. 6). Reaction time and accuracy of behavioral responses were recorded.

Experiment 2. The version used in the Experiment 2 was shorter, and without study phase. Total 60 trials were performed in two blocks of 30 trials each, with a short break between blocks. The working memory task lasted approximately 18 minutes overall. For each memory trial, a sample image with a green border was initially presented for 3 sec the participant indicated whether each of five successive test images matched or did not match the sample. A fixation with jittered delay (1.1–1.4 seconds) was placed between each test image. No scramble images were seen between trials as it was in Experiment 1 (Also see illustration and description of the task in Experiment 2 in a previous publication [47]).

### Behavioral data analysis

Accuracy of behavioral responses (number of correct responses), learning rate, and reaction times in milliseconds (msec) were determined for each object retrieval, new or studied item matching target, new or studied non-match distractors as previously described [2]. Exploratory analysis included the use of the descriptive statistics including means, standard deviations for continuous variables. Results were presented over the three repeated retrieval of memory target matches. Two sample t-tests were used to compare means and regression models were used to compare slopes All data analyses were performed in R (version 3.3.3).

## SUPPLEMENTARY MATERIAL FIGURE


