# Supplementary material for: Functional human GRIN2B promoter polymorphism and variation of mental processing speed in older adults
Source: Aging (Albany NY). 2017 Apr 24;9(4):1293–305. doi: 10.18632/aging.101228 (PMC5425128; doi:10.18632/aging.101228)
Supplement: Supplementary file 1 [file aging-09-1293-s001.pdf]

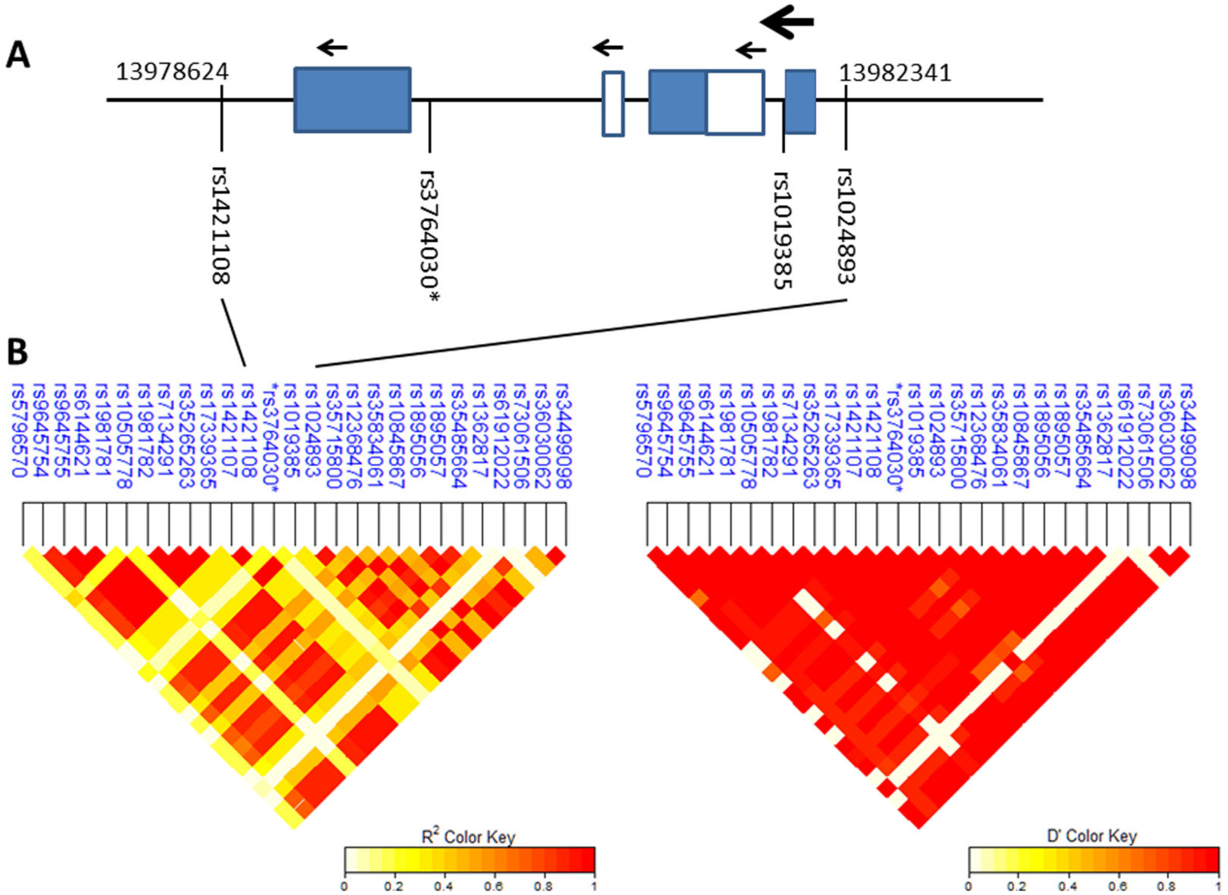

Figure S1. Panel (**A**) Schematic of the human *GRIN2B* gene showing locations of the first four non-coding exons and the 5' flanking region. SNP rs3764030 is shown with an asterisk. Boxes indicate the non-coding exons from *GRIN2B*. A major transcription start site in brain is shown by a large left-pointing arrow. Other transcription start sites are shown with smaller left-pointing arrows. The non-coding exons are known to undergo extensive alternative pre-mRNA splicing (shown as open boxes). Nucleotide positions from the reference sequence are shown above the horizontal line. Adapted from NCBI: www.ncbi.nlm.nih.gov RefSeqGene NG_031854.1. *GRIN2B* markers used in linkage equilibrium (LD) analysis are indicated by their dbSNP rs identifier. Orientation of the map is shown with transcription proceeding from right to left. Panel (**B**) Pairwise LD relationships for 27 markers in the CEU population with values shown in color below the *GRIN2B* gene map: R^2 (left) and D' (right). Bright red indicates a region of high LD ($R^2 > 0.8$, $D' > 0.8$) with yellow indicating areas of lower LD. The Lowest LD ($R^2 < 0.1$, $D' < 0.1$) are shown in white. Over the entire 20 kb region, R^2 was low while D' was high.
